# Assessing Liver Fibrosis Using 2D-SWE Liver Ultrasound Elastography and Dynamic Liver Scintigraphy with 99mTc-mebrofenin: A Comparative Prospective Single-Center Study

**DOI:** 10.3390/medicina59030479

**Published:** 2023-02-28

**Authors:** Donatas Jocius, Donatas Vajauskas, Artūras Samuilis, Kipras Mikelis, Skirmante Jokubauskiene, Kestutis Strupas, Algirdas E. Tamosiunas

**Affiliations:** 1 Department of Radiology, Nuclear Medicine and Medical Physics, Institute of Biomedical Sciences, Faculty of Medicine, Vilnius University, LT-01513 Vilnius, Lithuania; 2 Department of Radiology, Medical Academy, Lithuanian University of Health Science Kauno Klinikos, LT-44307 Kaunas, Lithuania; 3 National Center of Pathology, LT-01513 Vilnius, Lithuania; 4 Clinic of Gastroenterology, Nephro-Urology and Surgery, Institute of Clinical Medicine, Faculty of Medicine, Vilnius University, LT-01513 Vilnius, Lithuania

**Keywords:** chronic liver disease, liver fibrosis, ultrasound elastography, dynamic liver scintigraphy, 99mTc-mebrofenin

## Abstract

*Background and Objectives:* Many quantitative imaging modalities are available that quantify chronic liver disease, although only a few of them are included in clinical guidelines. Many more imaging options are still competing to find their place in the area of diagnosing chronic liver disease. We report our first prospective single-center study evaluating different imaging modalities that stratify viral hepatitis-associated liver fibrosis in a treatment-naïve patient group. *Materials and Methods:* The aim of our study is to compare and to combine already employed 2D shear wave elastography (2D-SWE) with dynamic liver scintigraphy with 99mTc-mebrofenin in chronic viral hepatitis patients for the staging of liver fibrosis. *Results:* Seventy-two patients were enrolled in the study. We found that both 2D-SWE ultrasound imaging, with dynamic liver scintigraphy with 99mTc-mebrofenin are able to stratify CLD patients into different liver fibrosis categories based on histological examination findings. We did not find any statistically significant difference between these imaging options, which means that dynamic liver scintigraphy with 99mTc-mebrofenin is not an inferior imaging technique. A combination of these imaging modalities showed increased accuracy in the non-invasive staging of liver cirrhosis. *Conclusions:* Our study presents that 2D-SWE and dynamic liver scintigraphy with 99mTc-mebrofenin could be used for staging liver fibrosis, both in singular application and in a combined way, adding a potential supplementary value that represents different aspects of liver fibrosis in CLD.

## 1. Introduction

Chronic liver disease (CLD) is a hallmark result of long-standing liver injury, with hepatitis virus infections, mainly hepatitis B and hepatitis C, being one of the leading causes and being responsible for almost 40% of CLD worldwide [1,2]. Other causes include NAFLD/NASH (nonalcoholic fatty liver disease/nonalcoholic steatohepatitis), alcohol, primary biliary cirrhosis, primary sclerosing cholangitis, α1 antitrypsin deficiency, Wilson’s disease, and autoimmune hepatitis [2]. Although there is a general trend of decreasing CLD mortality rates in the world, inequality between different regions is present [3]. In addition, the World Health Organization has stated that viral hepatitis infections have caused over one million deaths in 2019 [4].

### Aim

The aim of this prospective, non-randomized study is to compare already employed 2D-SWE ultrasound imaging with dynamic liver scintigraphy with 99mTc-mebrofenin in chronic viral hepatitis patients for the staging of liver fibrosis before initiating primary treatment. The histological evaluation of the hepatic tissue was used as a reference.

Multiple pathological factors affect the liver, with damage commonly resulting in liver fibrosis [5]. In general, pathogenic factors affecting the liver lead to the death of hepatocytes and the infiltration of immunogenic cells. Inflammatory alteration turns hepatic stellate cells into myofibroblasts—key cells for the production of collagen [6]. In the short term, this process is balanced and works to repair damaged liver tissue, while in chronic disease, the balance is disrupted and leads to an overproduction of the extracellular matrix [7,8].

In the early stages, liver fibrosis remains clinically silent most of the time, but on the other hand, as fibrosis progresses to its advanced stages, it alters liver function, leading to hepatic insufficiency and liver cirrhosis [9]. These alterations cause many devastating and life-threatening complications—portal hypertension and portosystemic varices, splenomegaly, ascites and spontaneous bacterial peritonitis, hepatorenal, hepatopulmonary syndromes, coagulopathy, bone-related diseases (osteopenia and osteoporosis), hematologic abnormalities, and liver cancer [10].

Clinical signs and symptoms such as jaundice, splenomegaly, ascites, gynecomastia, and others may not necessarily approach as the disease progresses [11]. Many patients without any clinical signs of liver damage may have been diagnosed with liver fibrosis via laboratory assays, imaging, or instrumental tests [12].

Once the diagnosis of CLD is present, a quantitative assessment of liver parenchyma is needed to stratify the risk of potential complications, deciding on the time and regime of treatment, and as a basic point for longitudinal follow-up [13,14].

Liver biopsy is historically the “golden” standard for directly assessing liver fibrosis stage, inflammatory activity, steatosis level, biliary disease, and other overlapping syndromes, and it is still incorporated into clinical guidelines [13,15]. On the other hand, liver biopsies are prone to many drawbacks, including their invasiveness, small sampling size, high interobserver and intraobserver variability, a limited availability for longitudinal follow-up, and the relatively high price and low accuracy [13,15].

At present, several imaging techniques are employed to quantitate diffuse hepatic tissue changes in patients with CLD. One of the most often used are ultrasound elastography with its several types, including transient elastography (TE), point shear wave elastography (pSWE), real time 2D shear wave elastography (2D-SWE), and to a lesser extent, magnetic resonance elastography (MRE) with several different sequences [16,17,18,19]. Nevertheless, both imaging and clinical guidelines suggest elastography as a method for ruling out advanced liver fibrosis rather than diagnosing it [15,16,20,21,22,23]. In addition, variable cutoff values measured with different vendor machines may overlap; thus, recommendations usually advise against using it in separating the exact fibrosis stage [24].

Computed tomography was also tested in quantitating chronic liver disease with several approaches, including liver surface nodularity or the liver segmental volume ratio, although software availability and the accuracy of these methods in the early stages of the disease are limited [18,25,26,27,28].

All of the abovementioned imaging techniques depict mechanical changes within the liver, which are associated with the amount of fibrotic tissue. Altered liver structural composition changes the liver’s mechanical properties, namely, elasticity and viscosity [29,30,31]. Measuring these mechanical deviations is a non-direct assessment of deteriorating liver function, and it is also related to several pitfalls and physiological states [13]. Liver stiffness measured via both USE and MRE could be elevated when liver inflammation, infiltrative liver disease, congestive heart disease, cholestasis, food ingestion, and physical activity are present without any liver fibrosis [15,24].

During the course of CLD, the advancing fibrosis makes the liver undergo structural changes, together with its functional deterioration [5]. To date, only scarce literature can be found on the role of functional imaging in CLD [32,33]. Nevertheless, functional imaging with several different nuclear medicine tracers was proven to represent liver function, predicting future remnant liver in major liver surgery [34,35,36,37,38].

Moreover, dynamic liver scintigraphy with 99mTc-mebrofenin showed to be as accurate as Gd-EOB-DTPA enhanced magnetic resonance imaging (MRI) when evaluating changes in liver function after portal vein embolization [36].

Our recently published data prove that dynamic liver scintigraphy with 99mTc-mebrofenin can stratify patients with CLD. Many scintigraphy parameters were found to be significant when predicting different liver fibrosis stages. Of all the calculated parameters, tracer retention in the liver and liver clearance were the most accurate ones [39]. Nevertheless, this modality still needs to be compared with the already established one.

## 2. Materials and Methods

During the period of August 2018 to January 2020, we prospectively invited patients who were referred to our center for a liver biopsy procedure as initial staging, to participate in this study and to undergo both imaging procedures and a liver biopsy. All agreeing participants have provided their informed consent. The study was approved by the local biomedical ethics committee (Vilnius Regional Biomedical Research Ethics Committee, Reg. No 158200-16-877-386), and the study was conducted according to the principles of the Helsinki Declaration.

Imaging procedures (2D-SWE and dynamic liver scintigraphy with 99mTc-mebrofenin) were performed on the same day. Patients were instructed to fast for at least 4 h before the procedure, and were advised against consuming coffee or energy drinks, or smoking. The imaging took place after a rest period of 10–15 min.

Liver ultrasound elastography 2D-SWE (GE Logiq E9 system, GE Healthcare, Wauwatosa, WI, USA) was performed using the convex probe in a supine position with the right arm elevated above the head. Measurements were performed in the right liver lobe, avoiding major blood vessels, and at least 1 cm away from liver capsule. Ten measurements were performed in each patient, and the results were expressed in kPa. Measurements within the interquartile range to median range (IQR/M) of less than 30% were accepted as being valid. The 2D-SWE imaging protocol was set according to Barr et al. [24]

Subsequently, on the same day, all patients underwent dynamic liver scintigraphy with 99mTc-mebrofenin on the GE Infinia Hawkeye dual head SPECT/CT gamma camera (GE Healthcare, Milwaukee, WI). Dynamic planar imaging was performed using low energy high resolution (LEHR) collimators (energy window 130–150 keV, matrix 64 × 64). The patient was placed in the supine position, and imaging was set immediately after an intravenous bolus injection of 99mTc-mebrofenin (Bridatec, GE Healthcare; median activity 205.5 MBq (SD ±14.15 MBq)) and continued for 30 min. The imaging protocol was adjusted according to Rassam et al. [38].

Data gathered were reconstructed using a GE Xeleris 2 workstation (General Electric Healthcare, Milwaukee, WI, USA). The geometric mean (Gmean) dataset was used for region of interest (ROI) placement.

Despite gathering the data of many dynamic liver scintigraphy parameters, in this comparison study, we used the liver clearance (LCL) of the right liver lobe, representing the amount of tracer extracted by the liver from the blood to the biliary system (a direct measurement of liver function). LCL was calculated according to Ekman et al. [40]. The LCL measurement was corrected for body surface area (BSA) and liver area (LA) to acquire a universal, patient-, and ROI size-independent result, and this was expressed in %/min/m^2^/dm^2^. The ROI of LCL was drawn on the right liver lobe, excluding the major biliary ducts and gall bladder.

Both imaging procedures were performed by the same investigator (D.J.) to maximize sampling of the same liver area, and to exclude interobserver variability.

Participants underwent a liver biopsy within two weeks after imaging studies. Liver biopsy procedures were performed using an 18-gauge biopsy needle to sample 2 or 3 biopsy cores in the right liver lobe to obtain a representative sample of at least 3 cm of total length. A histological examination was performed by an experienced pathologist (S.J.). METAVIR, hepatitis activity index (HAI) according to Ishak, and liver steatosis scores were evaluated.

The data were analyzed using Microsoft Excel (Microsoft Corporation, 2018), IBM SPSS Statistics (Version 25.0. Armonk, NY, USA: IBM Corp.), MedCalc Statistical Software (version 20.116. MedCalc Software bv, Ostend, Belgium), and Rstudio (version 2022.07.2. RStudio Team. RStudio: Integrated Development Environment for R. Boston, MA, USA).

All variables were tested for normality using the Shapiro–Wilk test; statistical tests were selected accordingly, and data were expressed as mean and standard error when normal data distribution was present, or in median and range if normal data distribution was not found.

We looked for associations between the fibrosis score from the core needle biopsy sample, using 2D-SWE and LCL scintigraphy parameters. Student’s t-test was used to find differences between two groups of variables with normal distribution, and the Mann–Whitney U test, for non-parametric continuous variables. For differences between three or more groups, a one-way analysis of variance (ANOVA) and the Kruskal–Wallis H test were used. Pearson’s correlation test and Spearman’s rank-order correlation coefficient were used to find correlations between continuous variables. A binomial logistic regression model was used to investigate whether a combination of independent variables predicted a probability of fibrosis stage. An R program, Compbdt, was used to compare sensitivity and specificity between tests for liver fibrosis (2D-SWE, LCL, or a regression model). A MedCalc function, Comparison of ROC curves, with the method of DeLong et al., was used to compare areas under the curve of these tests, and the Youden Index was used to set the threshold values [41,42]. The significance level was set at 0.05.

We calculated the sample size needed to achieve the desired statistical power for our study. For SWE, we used an estimate of AUC to be approximately 0.9, and predicted a 15 percent difference between the SWE and the scintigraphy measurements AUC. Then, using the estimation from Hajian-Tilaki, we calculated that a sample size of 70 patients was needed to achieve a 95% confidence level and 80% power [43].

## 3. Results

### 3.1. Patients

One hundred and six patients were invited to participate in the study, and 72 of them agreed to participate and signed informed consent forms. Imaging studies of both 2D-SWE and dynamic liver scintigraphy with 99mTc-mebrofenin were performed in all patients, and only one liver biopsy procedure was skipped due to an unrelated urgency precluding an invasive procedure (patient data was excluded from further analysis).

The mean patient age was 45 years (a range of 18–80 years). Sixty-eight participants had chronic hepatitis C (HCV), and four had chronic hepatis B (HBV). Nine patients also had a human immunodeficiency virus (HIV) coinfection.

The mean body mass was 81.89 kg (a range of 48–130 kg). The mean body surface area (BSA) (used for correction purposes) was 1.98 m^2^ (range: 1.45–2.52 m^2^) (Table 1).

All patients included in the study were treatment-naïve.

### 3.2. Histological Examination

The results of the histological examination are presented in Table 2. One should note that the histological analysis covers all but one patient who skipped a liver biopsy procedure. All of the other patients conceded to liver biopsy, and an analysis of biopsy specimens is available.

### 3.3. Imaging Studies

#### 3.3.1. Ultrasound Elastography: 2D-SWE Findings

**Figure 1:**
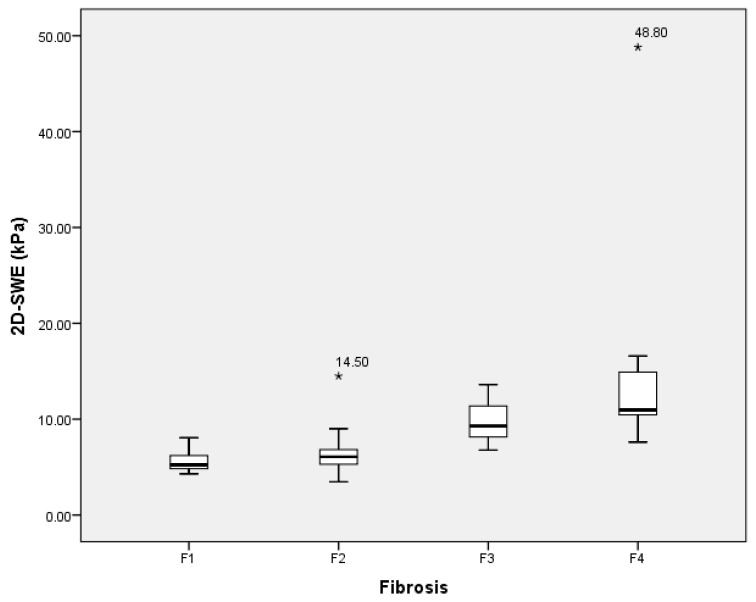
Box plot of the liver stiffness median, measured using 2D-SWE in different liver fibrosis categories according to METAVIR score. Median values: F1—5.23 kPa, F2—6.08 kPa, F3—9.28 kPa, and F4—10.97 kPa. 2D-SWE—multidimensional shear wave elastography.

The 2D-SWE was completed in all patients. There were no nondiagnostic examinations. The median liver stiffness in the study population was 6.73 kPa (range: 3.47 kPa–48.8 kPa). Liver fibrosis was evaluated via histological examination, patients were divided into four categories, and the liver stiffness median was calculated for each of these four categories (Figure 1).

The results of the histological examination were taken as a base value, and by relying on the histology grades of liver fibrosis, we calculated the 2D-SWE threshold values in different liver fibrosis stages (Table 3).

The 5.4 kPa value differentiated F1 (mild fibrosis) versus F2–F4. A threshold value of 7.16 kPa was set for differentiating F1–F2 versus F3–F4 (significant fibrosis), and a threshold value of 9.9 kPa was set for defining advanced fibrosis (F1–F3 versus F4).

#### 3.3.2. Dynamic Liver Scintigraphy with 99mTc-mebrofenin

**Figure 2:**
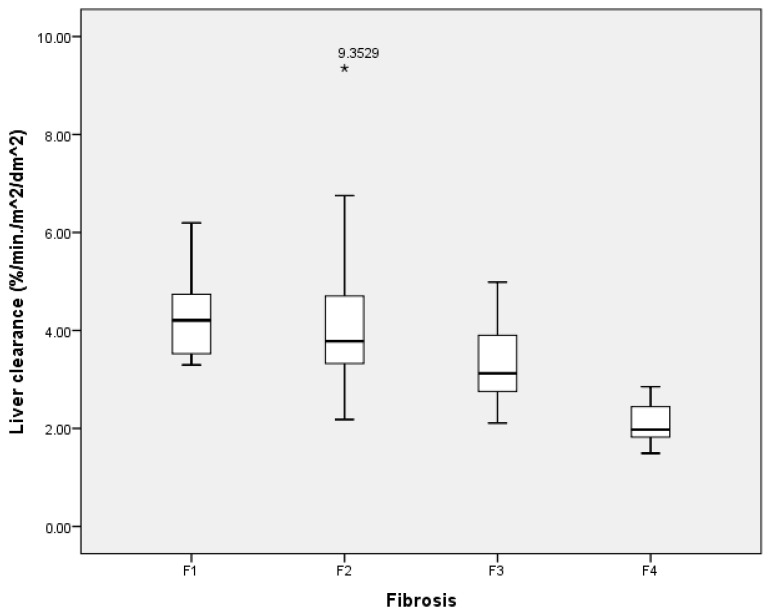
Box plot of the liver clearance (LCL) median, measured using dynamic liver scintigraphy with 99mTc-mebrofenin in different liver fibrosis categories according to the METAVIR score. Median values: F1—4.21%/min/m^2^/dm^2^; F2—3.78%/min/m^2^/dm^2^; F3—3.13%/min/m^2^/dm^2^; F4—1.98%/min/m^2^/dm^2^.

The liver clearance of the right lobe was selected because liver biopsies were only performed on the right liver lobe, and also as the only 2D-SWE measurement. The median LCL was 3.73%/min/m^2^/dm^2^ (range: 1.49%/min/m^2^/dm^2^–9.35%/min/m^2^/dm^2^); its results at different liver fibrosis stages are presented in Figure 2.

In general, liver clearance was negatively associated with the liver fibrosis stage—liver clearance decreased as the stage of liver fibrosis increased.

Threshold values for LCL were also calculated by relying on a histological examination of the liver biopsy specimen. The LCL of 3.76%/min/m^2^/dm^2^ differentiated F1 versus F2–F4, LCL of 3.29%/min/m^2^/dm^2^ clearance differentiated F1–F2 versus F3–F4, and an LCL of 2.85%/min/m^2^/dm^2^ was set as a point between F1–F3 and F4. The sensitivities, specificities, and AUROC of each threshold are presented in Table 4.

#### 3.3.3. Comparison between Imaging Studies

Both 2D-SWE and dynamic liver scintigraphy, with 99mTc-mebrofenin imaging methods, were able to separate different levels of liver fibrosis. A pathology examination of the liver biopsy specimen was used as a reference to compare liver stiffness (measured in kPa) and liver clearance (measured in %/min/m^2^/dm^2^) between the two imaging methods.

**Figure 3:**
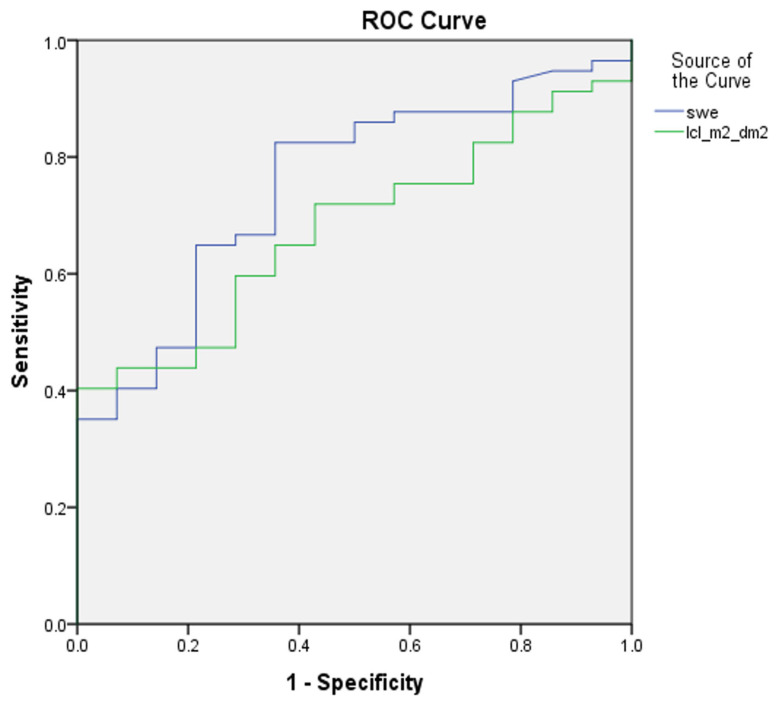
ROC curves of 2D-SWE and dynamic liver scintigraphy, with 99mTc-mebrofenin separating mild liver fibrosis; AUROC was 0.75 and 0.68, respectively (*p* value 0.22). swe—2D-SWE value represented in kPa; lcl_m^2^_dm^2^—liver clearance measured in right liver lobe and corrected using BSA and LA.

In defining mild versus advanced liver fibrosis (F1 vs. F2–F4), the sensitivity and specificity of 2D-SWE against dynamic liver scintigraphy with 99mTc-mebrofenin was 82% versus 59%, and 64% versus 71%, respectively. The AUROC was 0.75 for 2D-SWE, and 0.68 for dynamic liver scintigraphy with 99mTc-mebrofenin (*p* value 0.22) (Figure 3).

**Figure 4:**
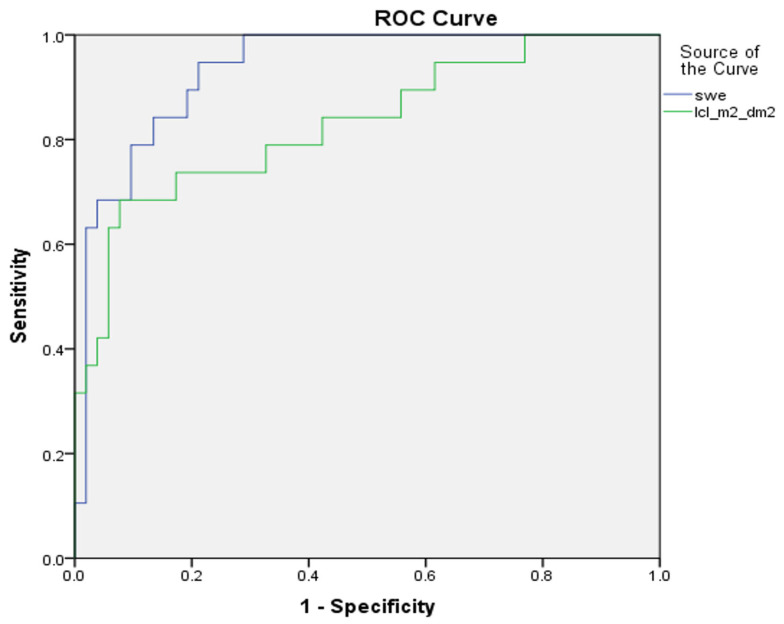
ROC curves of 2D-SWE and dynamic liver scintigraphy, with 99mTc-mebrofenin separating significant liver fibrosis; AUROC was 0.93 and 0.83, respectively (*p* value 0.061). swe—2D-SWE value represented in kPa; lcl_m^2^_dm^2^—liver clearance measured in the right liver lobe and corrected using BSA and LA.

In separating significant fibrosis (F1–F2 versus F3–F4), 2D-SWE and dynamic liver scintigraphy with 99mTc-mebrofenin showed sensitivities of 94% and 74%, and specificities of 79% and 83%, respectively. The AUROC was 0.93 for 2D-SWE, and 0.83 for dynamic liver scintigraphy, with 99mTc-mebrofenin (*p* value 0.061) (Figure 4).

**Figure 5:**
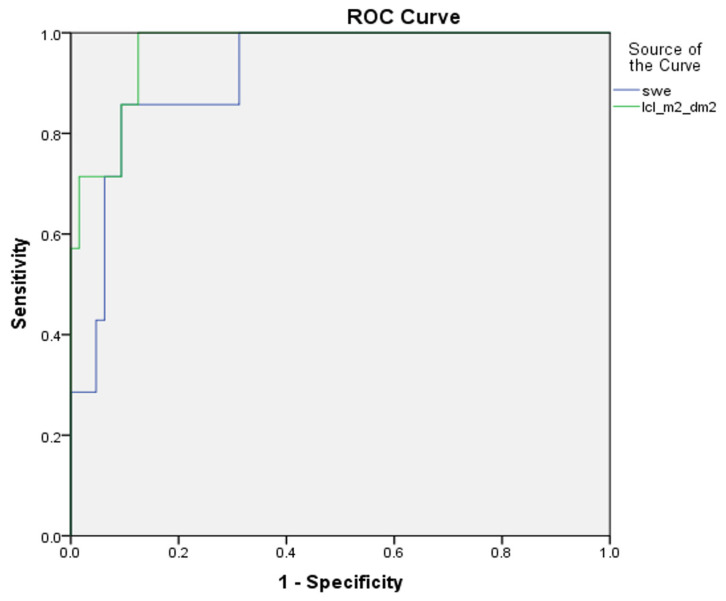
ROC curves of 2D-SWE and dynamic liver scintigraphy, with 99mTc-mebrofenin separating liver cirrhosis; AUROC was 0.91 and 0.96, respectively (*p* value 0.33). swe—2D-SWE value, represented in kPa; lcl_m^2^_dm^2^—liver clearance measured in right liver lobe and corrected using BSA and LA.

In separating advanced liver fibrosis/liver cirrhosis (F1–F3 versus F4), we found an 85% sensitivity and 85% specificity for 2D-SWE, and a 90% sensitivity and 87% specificity for dynamic liver scintigraphy with 99mTc-mebrofenin. The AUROC was 0.91 and 0.96, respectively (*p* value 0.33) (Figure 5).

### 3.4. Combination of Two Imaging Tests

The two imaging methods, 2D-SWE and dynamic liver scintigraphy with 99mTc-mebrofenin, represent in general somewhat different aspects of liver injury. The first one represents the change of the liver’s mechanical properties and the increasing amount of fibrotic tissue inside the liver, while the second method reflects alterations in liver function.

As these two methods may in theory be complementary to each other, we combined them in a logistic regression model to see whether this could have any additional value.

**Figure 6:**
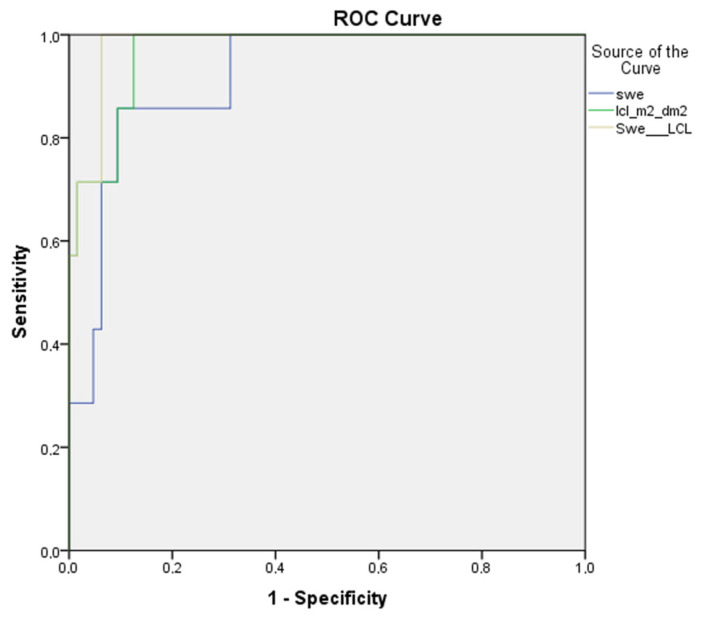
Combination of adding 2D-SWE and dynamic liver scintigraphy with 99mTc-mebrofenin together in liver cirrhosis group—the AUROC increased from 0.91 to 0.98. swe—2D-SWE curve before combination; lcl_m^2^_dm^2^—liver clearance measured in right liver lobe and corrected using BSA and LA curve before combination; SWE__LCL—combined curve.

Combining liver stiffness with liver clearance in separating mild (F1 versus F2–F4) and significant (F1–F2 versus F3–F4) fibrosis showed no difference in the area under the ROC curve—AUROC remained the same for mild (AUROC 0.75) and significant (AUROC 0.93) fibrosis, before and after the combination. Nonetheless, in liver cirrhosis (F1–F3 versus F4), the combination of the two methods increased imaging accuracy from AUROC 0.91 to 0.98, although the difference was non-significant (*p* value 0.18) (Figure 6).

## 4. Discussion

Many diagnostic assays have at least some value in diagnosing chronic liver disease, including simple laboratory and panel tests, noninvasive scoring systems, circulatory mRNA assays, several imaging modalities, and liver biopsies. Recently, artificial intelligence was also involved evaluating imaging findings and stratifying liver fibrosis [44,45,46]. Of note, only a few of these methods are included in clinical guidelines, while others are still competing to find their role in a CLD setting [12,15]

In general, ultrasound and magnetic resonance elastography are approved as valuable imaging tests for quantifying live fibrosis with high accuracy. Moreover, elastography techniques are rather used to rule out significant fibrosis in the low prevalence population. On the other hand, in a high prevalence patient population, guidelines suggest a cut-off to rule in significant fibrosis [15]. It is agreed that defining separate liver fibrosis stages with the presently recommended imaging modalities is not accurate enough, and is probably related to vendor dependency, in addition to physiological and pathological cofactors [16,20,24].

None of the biomarkers are excellent, and even the old “golden” standard—liver biopsy—has many of its own drawbacks related to procedural risk, incorrect evaluation due to sampling errors, a relatively small sample size, and high inter- and intra-observer variability [13,17]. The most widely used ultrasound elastography is also prone to several limitations, including vendor and observer variability, while cofounding pathological and physiological factors can lead to erroneous imaging conclusions [24]. In addition, shear wave elastography itself has several modifications, including transient elastography (TE), point shear wave (pSWE), and 2D shear wave elastography (2D-SWE), also estimating differences by measuring shear wave speed [20].

We note that dynamic liver scintigraphy with 99mTc-mebrofenin imaging has only scarce and indirect evidence in the CLD setting. Nevertheless, dynamic liver scintigraphy with 99mTc- mebrofenin for predicting future remnant liver functional volume is clearly valued and suggested over anatomical volumetry [38,47,48]. It is clear that healthy and abnormal liver parenchyma will have different functional capacities, which may not be present from structural imaging [49,50].

Our group has recently published data indicating that dynamic liver scintigraphy with 99mTc-mebrofenin may separate the different stages of liver fibrosis [39]. Together with other partly related studies using 99mTc-mebrofenin to quantitate liver parenchyma, this has led our group to test this modality in the CLD setting by comparing it with an already employed method—2D-SWE [37,47,49,50].

To our knowledge, this is the first prospective study comparing 2D-SWE imaging with dynamic liver scintigraphy with 99mTc-mebrofenin that uses a histological examination of the liver biopsy specimen as a reference standard in the same group of treatment-naïve patients with chronic viral hepatitis.

**Figure 7:**
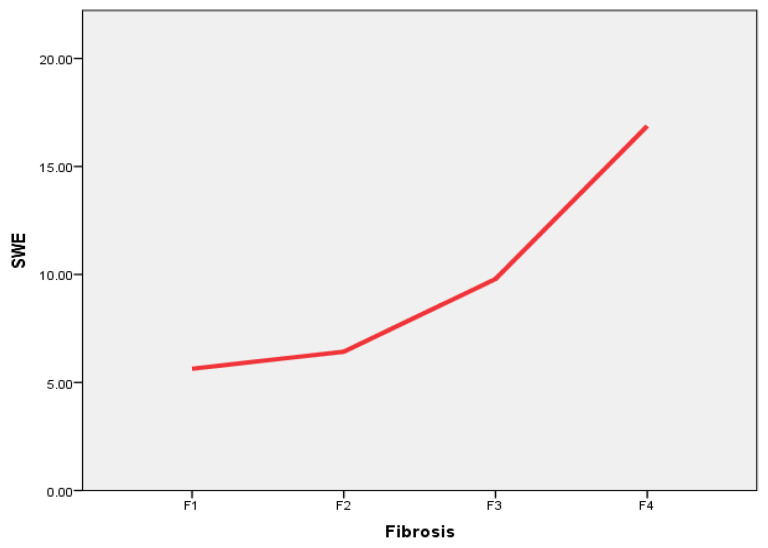
The 2D-SWE correlation with increasing liver fibrosis stage. Direct positive association is seen between liver fibrosis stage according to METAVIR and liver stiffness (2D-SWE), measured in kPa. SWE—2D-SWE value represented in kPa; Fibrosis—liver fibrosis evaluated in liver biopsy specimens according to METAVIR.

**Figure 8:**
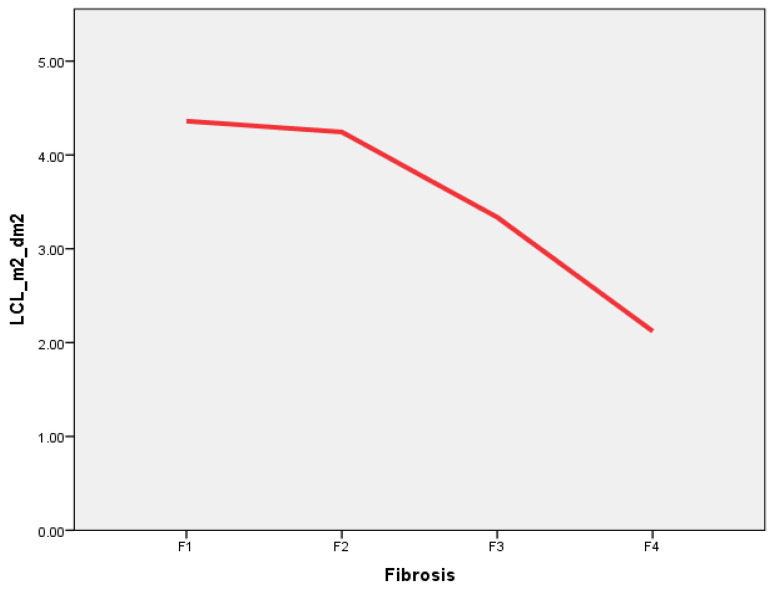
Liver clearance (LCL) relation to increasing liver fibrosis stage. Direct negative association between liver clearance (measured in %/min/m^2^/dm^2^), and liver fibrosis stage measured according to METAVIR is present. LCL_m^2^_dm^2^—liver clearance measured in right liver lobe and corrected with BSA and LA; Fibrosis—liver fibrosis evaluated in liver biopsy specimen according to METAVIR.

Several conclusions could be stated. First, we found that both liver stiffness (measured using 2D-SWE in kPa) and LCL (measured via dynamic liver scintigraphy with 99mTc-mebrofenin in %/min/m^2^/dm^2^) resemble liver changes at different stages of liver fibrosis. Liver stiffness had a direct positive association with liver fibrosis—liver stiffness increased as fibrosis developed (Figure 7). On the other hand, LCL had a direct negative association to liver fibrosis—it decreased as liver fibrosis developed (Figure 8). The difference in median values between the liver fibrosis stages in each imaging value was significant (Figure 1 and Figure 2).

The 2D-SWE findings reflect the underlying pathological mechanical changes of the liver as an indirect evidence of liver tissue injury. Liver stiffness increases with the increasing amount of fibrotic tissue in the liver parenchyma during CLD, and as the disease progresses, the amount of fibrotic tissue also expands [51,52]. On the other hand, LCL represents functional alterations when CLD progress, making it clear that liver clearance decreases with increasing liver fibrosis. This finding can be explained on a molecular level—99mTc-mebrofenin is an iminodiacetic acid derivate, a lidocaine analog, which is taken up by hepatocytes from the blood through organic anion transporting polypeptide (OATP) receptors on the cell membrane, and excreted into bile canaliculi without any metabolism [38,53]. During the course of CLD, hepatocytes undergo structural alterations, while, together with other changes, the number of OATP receptors decreases; in turn, the uptake of iminodiacetic acid derivates, such as mebrofenin, is also decreased [54,55].

Second, in comparing 2D-SWE and dynamic liver scintigraphy with 99mTc-mebrofenin, we found no significant difference in AUROC at any of liver fibrosis stages (see Figure 3, Figure 4 and Figure 5), although the numerical value of the area under ROC curve was somewhat higher for liver stiffness in mild and significant fibrosis. On the other hand, in liver cirrhosis, AUROC was higher for liver clearance. These findings support dynamic liver scintigraphy with 99mTc-mebrofenin as a non-inferior imaging method for stratifying CLD.

Third, both imaging methods work by somewhat different mechanisms—2D-SWE represents mechanical changes in the liver tissue by evaluating the speed of transverse soundwaves propagating in altered hepatic parenchyma [29,30]. A different approach is employed in dynamic liver scintigraphy with 99mTc- mebrofenin. The passage of the iminodiacetic acid derivate mebrofenin through OATP receptors situated on the hepatocyte basolateral membrane resembles liver cell function, which is altered as liver fibrosis progresses [53,54]. By referring a different approach to these quantitative liver imaging modalities, we also combined them together to check if there is additional value. Although no additional value was seen in mild and significant fibrosis, at the late stages of CLD (liver cirrhosis), the area under the ROC curve increased from 0.91 to 0.98 (see Figure 6), which signifies an increased accuracy when two imaging modalities are combined.

Several drawbacks and issues of the present study are relevant. This was a single-center, non-blinded study. Its relatively small sample size may hamper a deeper investigation of our study results, especially in some subgroups with particularly small sizes. In addition, the diversity of CLD etiology, including both HBV and HCV patients, as well as some with HIV infections, may pose additional conflict in interpreting results.

The reference standard liver biopsy has its well-known limitations, adding that it does not represent the heterogeneity of liver fibrosis, especially when compared to quantitative imaging, where larger areas of the liver parenchyma are sampled—relying on liver biopsy by itself could be erroneous.

Both imaging and liver biopsy were performed by a single operator, posing potential bias and precluding a comparison of interobserver agreement.

All imaging measurements were performed on a small area of the right liver lobe, which hindered the evaluation of larger volumes and overcoming the possibility of tissue heterogeneity.

## 5. Conclusions

To conclude, our study results showed that both 2D-SWE and dynamic liver scintigraphy with 99mTc-mebrofenin can stage liver fibrosis. We want to emphasize that according to our results, we found no statistically significant differences between these tests. On the contrary, we found that by leaning on the different mechanisms used by these imaging approaches, they can be combined to increase diagnostic accuracy, as we have demonstrated, in the late stages of liver fibrosis.

Indeed, this should be clarified in a wider and multicenter manner, although our preliminary results do look promising.

## Figures and Tables

**Table 1:** Demographic data of study population.

Variable	Value
Age (years)	45 years (SD 13.4)
Gender	
Male	45 (62.5%)
Female	27 (37.5%)
Weight (kg)	82.28 kg (SD 17.6)
BMI (kg/m^2^)	26.5 kg/m^2^ (17.51–42 kg/m^2^)
BSA (m^2^)	1.98 m^2^ (SD 0.23)
Virus type in patient population	
HBV	4 (5%)
HCV	68 (95%)
HIV coinfection	9 (12%)

BMI—body mass index, BSA—body surface area, HBV—hepatitis B virus, HCV—hepatitis C virus, HIV—human immunodeficiency virus.

**Table 2:** Distribution of liver fibrosis stages in the study population according to pathological examination.

	F1	F2	F3	F4
METAVIR	14 (19.4%)	38 (52.8%)	12 (16.7%)	7 (9.7%)

**Table 3:** 2D-SWE threshold values defining different liver fibrosis stages.

Liver Fibrosis	2D-SWE Value (kPa)	AUROC	Sensitivity (%)	Specificity (%)
F1 vs. F2-F4	5.4 kPa	0.75	82%	65%
F1-F2 vs. F3-F4	7.16 kPa	0.93	89%	79%
F1-F3 vs. F4	9.9 kPa	0.91	85%	91%

**Table 4:** Liver clearance threshold values defining different liver fibrosis stages.

Liver Fibrosis	LCL (%/min/m^2^/dm^2^)	AUROC	Sensitivity (%)	Specificity (%)
F1 vs. F2–F4	3.76	0.67	71%	60%
F1–F2 vs. F3–F4	3.29	0.83	82%	74%
F1–F3 vs. F4	2.85	0.96	87%	99%

## Data Availability

The datasets used and/or analyzed during the current study are available from the corresponding author upon reasonable request.

## Abbreviations

2D-SWE	two-dimensional shear wave elastography
99mTc	metastable nuclear isomer of technetium-99
ANOVA	a one-way analysis of variance
AUROC	area under receiver operator characteristics curve
BMI	body mass index
BSA	body surface area
CLD	chronic liver disease
CT	computed tomography
Gd-EOB-DTPA	gadolinium ethoxybenzyl diethylenetriaminepentaacetic acid
HBV	hepatitis B virus
HCV	hepatitis C virus
HIV	human immunodeficiency virus
kPa	kilopascal
LA	liver area
LCL	liver clearance
LEHR	low energy high resolution
METAVIR	meta-analysis of histological data in viral hepatitis
MRE	magnetic resonance elastography
MRI	magnetic resonance imaging
mRNA	messenger ribonucleic acid
NAFLD	nonalcoholic fatty liver disease
NASH	nonalcoholic steatohepatitis
OATP	organic anion transporter polypeptide
pSWE	point shear wave elastography
ROC	receiver operator characteristics curve
ROI	region of interest
SPECT	single photon emission computed tomography
TE	transient elastography
USE	ultrasound elastography
